# The Medical Letter Bag

**Published:** 1919-04

**Authors:** 


### The Medical Letter-Bag.

There is every reason why The Medical Letter-Bag should become one of the best departments of the Journal if the physicians will only be unselfish enough to give others the benefit of their experiences or observations. Every doctor who has an unusual case of any kind should feel enough interest in humanity to let other doctors have the benefits of his practice and observations in the treatment of the case. In this way members of the profession can be of great help to each other. An interesting question is asked this month by Dr. J. L. DuBose. Perhaps you can answer it.

